# Power Play of Commensal Bacteria in the Buccal Cavity of Female Nile Tilapia

**DOI:** 10.3389/fmicb.2021.773351

**Published:** 2021-11-16

**Authors:** Yousri Abdelhafiz, Jorge M. O. Fernandes, Erika Stefani, Davide Albanese, Claudio Donati, Viswanath Kiron

**Affiliations:** ^1^Faculty of Biosciences and Aquaculture, Nord University, Bodø, Norway; ^2^Unit of Computational Biology, Research and Innovation Centre, Fondazione Edmund Mach, San Michele all’Adige, Italy

**Keywords:** Nile tilapia, buccal cavity, bacteria, 16S rRNA gene sequencing, commensal

## Abstract

Fish are widely exposed to higher microbial loads compared to land and air animals. It is known that the microbiome plays an essential role in the health and development of the host. The oral microbiome is vital in females of different organisms, including the maternal mouthbrooding species such as Nile tilapia (*Oreochromis niloticus*). The present study reports for the first time the microbial composition in the buccal cavity of female and male Nile tilapia reared in a recirculating aquaculture system. Mucus samples were collected from the buccal cavity of 58 adult fish (∼1 kg), and 16S rRNA gene amplicon sequencing was used to profile the microbial communities in females and males. The analysis revealed that opportunistic pathogens such as *Streptococcus* sp. were less abundant in the female buccal cavity. The power play of certain bacteria such as *Acinetobacter*, Acidobacteria (GP4 and GP6), and Saccharibacteria that have known metabolic advantages was evident in females compared to males. Association networks inferred from relative abundances showed few microbe–microbe interactions of opportunistic pathogens in female fish. The findings of opportunistic bacteria and their interactions with other microbes will be valuable for improving Nile tilapia rearing practices. The presence of bacteria with specific functions in the buccal cavity of female fish points to their ability to create a protective microbial ecosystem for the offspring.

## Introduction

The buccal cavity harbors a complex and diverse microbiota, and parallels can be drawn between human oral and gut bacterial communities (Maki et al., 2021). Although the oral microbiome composition is different between individuals and there exist differences between their micro-habitats, the principal function of the microbiome remains the same (Caselli et al., 2020). Microbial communities play a vital role in the physiological functions, immune system, and growth of the host.

The potential beneficial/commensal microbes of the oral cavity are essential for the wellbeing of the host. The composition of the oral microbiome in healthy individuals is generally stable. Imbalance in the microbial community is known as dysbiosis, and this condition can be associated with diseases (Zaura et al., 2009; Caselli et al., 2020; Su et al., 2020; Wynne et al., 2020). Although the composition of the oral microbiome changes with the host health status (as observed in the case of adolescence-related depression and anxiety), its diversity remains the same (Simpson et al., 2020). Pregnant women harbor more oral cavity microbes than non-pregnant women, and pathogenic taxa proliferate during early periods of pregnancy (Fujiwara et al., 2017; Lin et al., 2018). Such periodontal pathogens can cause oral diseases, which in turn can complicate the pregnancy and lead to adverse outcomes (Farrell et al., 2006; Salih et al., 2020; Saadaoui et al., 2021). Moreover, it is now known that chewing of betel-areca preparations and the use of tobacco and alcohol can have cytogenetic effects, jeopardize oral health and shift the microbial population; these health-risk factors are linked to oral cancer (Wang et al., 2019; Su et al., 2020).

As mentioned above, the association of the oral microbiome and the health of humans is well studied compared to those in fish. Hence, more information about the symbionts of fishes is essential because dysbiosis may also occur in the fish mouth and may cause a natural outbreak of various diseases (Wynne et al., 2020). To our knowledge, only one study has reported the importance of microbiome balance in the buccal cavity of a fish; a dysbiosis event that occurred in Atlantic salmon (*Salmo salar*) was linked to yellow mouth disease (Wynne et al., 2020).

Nile tilapia (*Oreochromis niloticus*) is a preferred farmed species because it exhibits excellent growth and robustness under culture conditions. Wild Nile tilapia are sexually mature when they attain a total length of 20–30 cm (Gómez-Márquez et al., 2003; Shoko et al., 2015). However, under captivity, sexual maturity is reached at a relatively smaller size of 8–13 cm (Gómez-Márquez et al., 2003; Shoko et al., 2015). Nile tilapia is a mouthbrooder species, and the females protect the eggs by incubating them in their mouth until hatching (Konstantinidis et al., 2020). This form of parental care increases offspring survival and fitness; the epidermal mucus of female tilapia changes to ensure protection, development and capacity enhancement of the embryos/fry under different situations, for example, during transport to new locations/environments (Iq and Shu-Chien, 2011; Orlando et al., 2017). Buccal cavity mucus of female tilapia has an array of proteins, namely antioxidant enzymes such as peroxiredoxin and stress proteins like heat shock proteins that are upregulated during infection and parental care (Iq and Shu-Chien, 2011). A possibility of passive immune transfer from mother to offspring during mouthbrooding rather than via eggs has been reported, based on a higher survival rate against ectoparasites compared to those raised through artificial incubation (Subasinghe, 1993; Sin et al., 1994).

There is growing evidence that the microbiome can be horizontally or vertically transmitted from mother to infant (Ferretti et al., 2018) and from parent fish to progeny (Sylvain and Derome, 2017). Hence, we wanted to understand the differences in buccal cavity microbiome profiles in female and male Nile tilapia to understand if the mouthbrooders have specific microbes to protect their offspring.

## Materials and Methods

### Ethics Statement

The study was conducted after obtaining the license from the Norwegian Animal Research Authority (FOTS ID 1042). The guidelines for research using experimental animals were strictly followed during Nile tilapia rearing, handling and tissue sampling.

### Experimental Fish and Set Up

In the present experiment, we employed Nile tilapia that were the offspring of the fish obtained by hatching eggs from wild fish that were captured from the Nile River, Luxor, Egypt (location GPS: 25°39′56″ N, 32°37′07″ E). The stocking density was 27 fish/m^3^, and the fish were reared in a freshwater recirculating system in a tilapia rearing facility at the Research Station of Nord University, Bodø, Norway. The rearing conditions were: dissolved oxygen – 8.33 mg/l, ammonia – 0.06 mg/l, nitrite – 0.03 mg/l, alkalinity – 53.92 mg/l as CaCO_3_, water temperature – 29.3 ± 0.4°C, photoperiod – LD 13:11. The fish were fed commercial pellets (Skretting, Norway) during the rearing period (Podgorniak et al., 2019). We collected sexually mature males (*n* = 30) and females (*n* = 28) (both of average weight 1000 g, average total length 37.48 cm, 8 month-old) from the above mentioned stock by carefully distinguishing them based on the tapered shape or rounded shape below the anus. The sex of the fish was further confirmed by dissection and observation of the gonads.

Prior to sampling, fish were not fed for 48 h, and they were sacrificed by exposing them to an emulsion containing 12 mL of clove oil (Sigma-Aldrich, St. Louis, MO, United States), 96% ethanol (1:10 v/v) and 10 L of water (Konstantinidis et al., 2020). Mouth mucus samples from the buccal cavity were taken using swabs (Copan Italia, Brescia, Italy), which were transferred to cryotubes and immediately frozen in liquid nitrogen (Caselli et al., 2020; Wynne et al., 2020). The collected samples were stored at −80°C until further use.

### Microbial DNA Extraction and Library Preparation

Each individual swab sample was transferred to a 5 ml tube containing 1.4 mm zirconium oxide beads (Cayman Chemical, Ann Arbor, MI, United States), and two ml of InhibitEX buffer (Qiagen, Hilden, Germany) were added into the tube. DNA was extracted using QIAamp DNA stool Mini Kit (Qiagen) according to the manufacturer’s protocol. The extracted DNA was eluted in 75 μl ATE buffer. Then, the quality and quantity of the extracted DNA were checked with the NanoDrop spectrophotometer ND-8000 (Thermo Fisher Scientific Inc., Waltham, MA, United States).

Library preparation was performed under sterile conditions. The 16S rRNA gene library was constructed from the extracted DNA using the specific bacterial primers 341F (5′ CCTACGGGNGGCWGCAG 3′) and 805R (5′ GACTACNVGGGTWTCTAATCC 3′) (Klindworth et al., 2013) flanked by overhang Illumina adapters targeting the hypervariable V3-V4 region (∼ 460 bp). The primer concentration was 10 nM and 1 μl was used for the library preparation. PCR reactions were prepared (25 μl total volume) using (12.5 μl) AmpliTag gold Master Mix (Thermo Fisher Scientific Inc.) and 2.5 μl of DNA template (5 ng/μl). PCR conditions consisted of an initial denaturation step at 95°C for 10 min (1 cycle), 30 cycles at 95°C for 30 s, 57°C for 30 s, 72°C for 1 min, and a final extension step at 72°C for 7 min (1 cycle).

Agarose gel (1.5%, 4.5 g/300 ml) electrophoresis was employed to check the amplified products. The purified PCR products obtained using the CleanNGS system (CleanNA, Waddinxveen, Netherlands) were subjected to a second PCR (8 cycles, 16S Metagenomic Sequencing Library Preparation, Illumina, San Diego, CA, United States). CleanNGS system (CleanNA) was used to purify the obtained amplicon libraries. The quality of the libraries was checked on a TapeStation 2200 platform (Agilent Technologies, Santa Clara, CA, United States). Thereafter, the libraries were quantified using the Quant-IT PicoGreen dsDNA assay kit (Thermo Fisher Scientific) on a Synergy 2 microplate reader (BioTek, Winooski, VT, United States). Next, the pooled libraries were quantified using the KAPA library quantification kit (Roche, Basel, Switzerland) on a real-time qPCR LightCycler 480 (Roche). They were then sequenced on an Illumina^®^ MiSeq (PE300) platform (MiSeq Control Software 2.5.0.5 and Real-Time Analysis software 1.18.54.0).

### Data Processing and Analyses

The generated paired-end reads were truncated at 270 bp using VSEARCH (Rognes et al., 2016), and then processed using MICCA pipeline (v1.7.2) (Albanese et al., 2015). Sequences with a minimum overlap length of 60 bp and a maximum mismatch of 20 bp were merged. Next, the forward and reverse primers were trimmed off the merged reads and reads that did not contain the primers were discarded. Thereafter, the sequences with an expected error rate (Edgar and Flyvbjerg, 2015) >0.75 were filtered out, and sequences shorter than 400 bp were discarded. The filtered reads were denoised using the “*de novo* unoise” method implemented in MICCA, which utilizes the UNOISE3 algorithm (Edgar, 2016). The denoising method, which is based on correcting sequencing errors and determining true biological sequences at single-nucleotide resolution, generates amplicon sequence variants (ASVs). The taxonomic assignment of the representative bacterial ASVs was performed using the RDP classifier (Lan et al., 2012). The sequences were aligned using the NAST (DeSantis et al., 2006) multiple sequence aligner, and a phylogenetic tree was prepared using the FastTree software available in the MICCA pipeline.

### Statistical Analysis

The similarities/differences in α-diversity were checked by Wilcoxon rank-sum test. Bacterial β-diversity was determined using unweighted and weighted UniFrac distances (Lozupone and Knight, 2005). Differences between bacterial communities in male and female groups were visualized by Principal Coordinates Analysis (PCoA). After checking the dispersions within the data set of each group, statistically significant differences between the groups were assessed using Permutational Multivariate Analysis of Variance Using Distance Matrices (PERMANOVA) (Anderson, 2001) (with 9,999 permutations), implemented in adonis function of the vegan R-package (Oksanen et al., 2013). The DESeq2 (Love et al., 2014) package was employed to detect the differentially abundant ASVs in the non-rarefied data (McMurdie and Holmes, 2014). It is believed that rarefied data reduce statistical power and make it difficult to assess the differences in the actual composition (Weiss et al., 2017).

### Microbial Network Analysis

Microbial communities are complex, and their function and structure are greatly influenced by microbe-host and microbe-microbe interactions. To investigate the latter connections, we calculated pairwise relationships from the relative abundances of ASVs associated with the two types of samples (females and males). The networks were constructed at the phylum level using the SpiecEasi package, which considers an inverse covariance matrix and conditional independence (Kurtz et al., 2015). The differences in degrees and betweenness of nodes in the network of female and male fish were checked using the Wilcoxon rank-sum test.

## Results

### Microbial Composition

To characterize the microbial composition in the buccal cavity of female and male Nile tilapia, the fish were reared in a common garden. The environmental conditions and diet that are known to affect the microbiota were kept constant throughout the experimental period. The amplicon sequencing of the 16S rRNA libraries generated 8706006 high-quality reads with an average of 150104 reads per sample. The reads were rarefied to 53575 reads per sample (without replacement), to take the read count variation in the different samples into account. A total of 1367 denoised ASVs were identified across all samples. Their taxonomic classification revealed the presence of bacteria belonging to 26 phyla and 272 genera.

**FIGURE 1:**
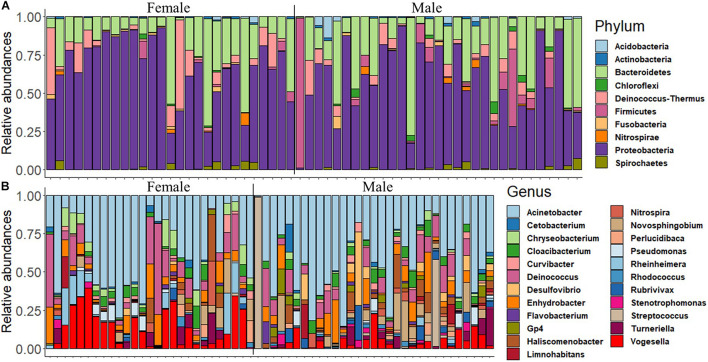
The relative abundance of the microbial composition in the buccal cavity of female and male Nile tilapia. **(A)** Top 10 dominant phyla. **(B)** Top 23 dominant genera.

First, we delineated the microbial composition in the mouth of female and male fish, and then we investigated the abundance of the dominant ASVs/taxa in females and males. The analysis revealed that Proteobacteria, Bacteroidetes, Firmicutes, Deinococcus–Thermus, Actinobacteria, and Acidobacteria were the most dominant phyla in both groups (Figure 1A and Supplementary Figure 1A). The dominance of Proteobacteria was also reflected in the microbial composition at the genus level, i.e., *Acinetobacter*, *Enhydrobacter*, *Novosphingobium*, *Pseudomonas*, *Haliscomenobacter*, *Rheinheimera*, and *Vogesella* (Figure 1B). However, the abundance of certain dominant genera such as *Acinetobacter* and *Enhydrobacter* was higher in females compared to males (Supplementary Figure 1B).

The proportions of the most dominant phyla and genera in both sexes are provided in Table 1. The proportions of Proteobacteria were 29.95 and 35.71% in females and males, respectively. The corresponding values of the phylum Bacteroidetes were 10.20 and 6.64%. Furthermore, *Acinetobacter* was the most abundant genus in the buccal cavity of female and male fish. The abundance of this genus in females was 28.07% compared to 26.19% males. The proportions (females vs. males) of the other genera belonging to the phylum Proteobacteria were: *Enhydrobacter* (5.01% vs. 2.87%), *Novosphingobium* (1.19% vs. 2.98%), *Pseudomonas* (1.37% vs. 3.15%), *Haliscomenobacter* (0.4% vs. 0.2%), *Rheinheimera* (0.99% vs. 4.83%), and *Vogesella* (4.57% vs. 8.80%).

**TABLE 1:** The proportion (%) of different bacteria in the buccal cavity of female and male Nile tilapia.

**Taxa**	**Female**	**Male**
**Phyla**		
Proteobacteria	29.95	35.71
Bacteroidetes	10.20	6.64
Firmicutes	0.10	3.47
Deinococcus–Thermus	1.18	3.13
Nitrospirae	0.48	3.13
Actinobacteria	0.67	1.23
Acidobacteria	1.76	0.97
Fusobacteria	0.32	0.30
**Genera**		
*Acinetobacter*	28.07	26.19
*Turneriella*	1.18	0.54
*Vogesella*	4.57	8.80
*Pseudomonas*	1.37	3.15
*Enhydrobacter*	5.01	2.87
*Rheinheimera*	0.99	4.83
*Novosphingobium*	1.19	2.98
*Streptococcus*	0.01	3.02
*Chryseobacterium*	2.21	2.93

**FIGURE 2:**
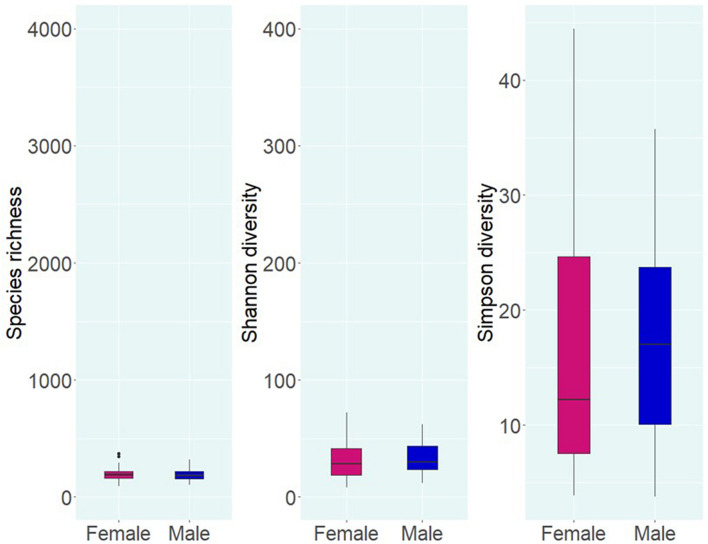
Alpha diversity of the bacteria in the buccal cavity of female and male Nile tilapia. Species richness, Shannon diversity, and Simpson diversity of the groups are not significantly different. The boxplots show minimum, lower quartile, median, upper quartile, and maximum values.

**FIGURE 3:**
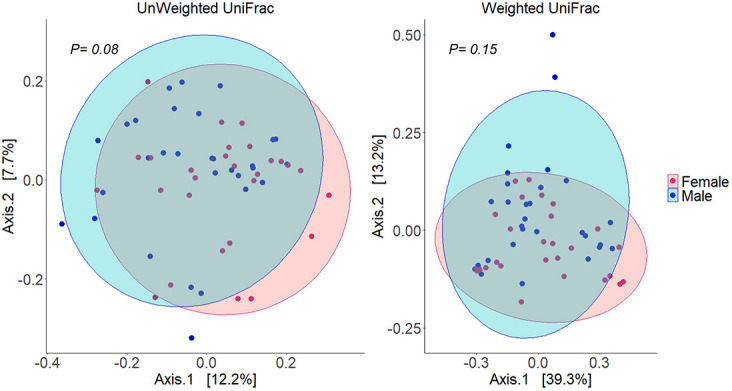
Principal coordinates analyses (PCoA) using distance (Unweighted and weighted UniFrac) matrices of the bacteria in the buccal cavity of female and male Nile tilapia. The ellipses were generated assuming that the data are from a multivariate normal distribution.

Alpha diversity analysis of microbial communities in female and male buccal cavities was based on three ecological diversity measures, namely, the Chao1 estimator of the number of species, which is a measure of richness, the Shannon diversity, which measures the evenness of the microbial populations, and the Simpson diversity, which measures the dominant species (Marcon and Hérault, 2015; Hsieh et al., 2016). Wilcoxon rank-sum test did not detect any statistical differences in species richness (*P* = 0.75), microbial evenness (*P* = 0.48), and dominant species (*P* = 0.55) of the microbial communities in the two groups (Figure 2). Beta diversity analysis also did not reveal the differences between the microbial communities in the two groups, based on PCoA and PERMANOVA test using both weighted and unweighted UniFrac distances (*P* > 0.05) (Figure 3). In the case of unweighted UniFrac distance, we observed a statistical trend (*P* = 0.08) that could be indicating a difference between rare microbial communities in female and male buccal cavities.

### Differential Abundance of Amplicon Sequence Variants Present in Female and Male Tilapia

**FIGURE 4:**
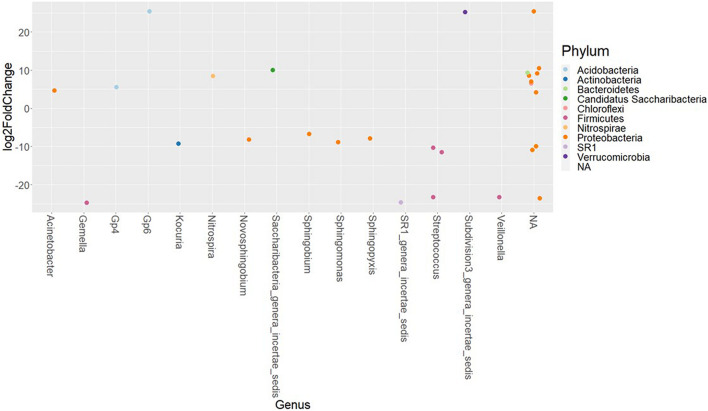
The significantly abundant amplicon sequence variants in the buccal cavity of female Nile tilapia compared to males. The *X*-axis labels are genus-level annotations of the microbes identified in the buccal cavity.

The differences in the abundances of ASVs of the buccal cavity samples of female and male Nile tilapia were evaluated employing the Wald-test in DESeq2. The results revealed significant differences between the two groups. The abundance of many opportunistic pathogens such as *Streptococcus, Gemella, Veillonella*, *Kocuria*, and *SR1*, which belong to *Firmicutes*, *Actinobacteria*, and *SR1* was found to be significantly lower in the female buccal cavity compared to that in male tilapia; fold changes ranged between −10 and −25 (Figure 4). On the other hand, the abundance of *Acinetobacter* that belongs to the phylum Proteobacteria was five-fold higher in female tilapia. Furthermore, the abundance of *Nitrospira* was nine-fold higher in females (Figure 4). Acidobacteria Gp6 and Gp4 had significantly higher abundance (25-fold and five-fold, respectively) in females compared to males. *Saccharibacteria* was also abundant (10-fold) in females compared to males.

### Microbial Network

**FIGURE 5:**
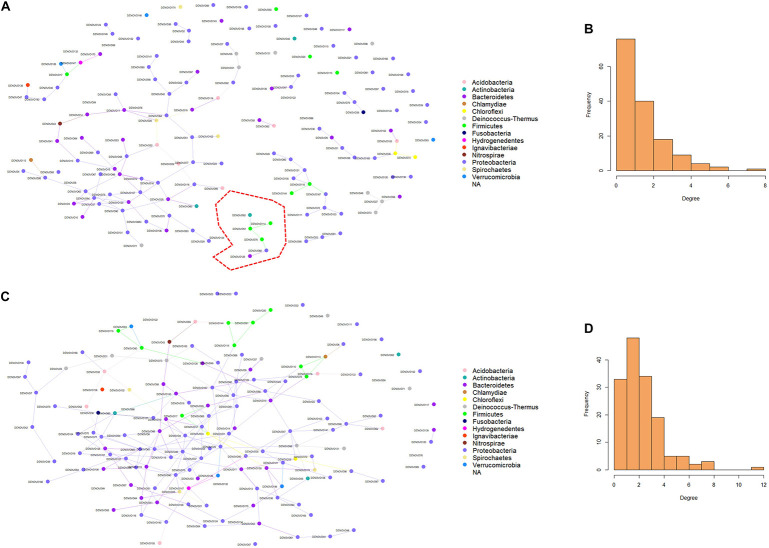
Predicted association of bacteria in the buccal cavity of female and male Nile tilapia. Network of bacteria in the female **(A)** and male **(C)** buccal cavity was constructed using the SpiecEasi package. Each node represents a taxon (ASV) and connections between nodes are shown using edges. The degree distribution in the network of bacteria of the female **(B)** and male **(D)** buccal cavity indicates the high node degree in the males **(D)**. The cluster in red is detailed in Figure 6.

Microbial networks were generated using the information from the abundance of the 150 dominant ASVs in both females and males. The microbial connections between the nodes in the networks were different in females and males (Figures 5A,C). The ASVs in female fish had fewer connections compared to males. These results are evident in the degree histograms (Figures 5B,D). Wilcoxon rank-sum test revealed that the node degrees as well as betweenness in female and male fish were significantly different, with a *P*-value < 0.05. In a network, each node has a degree which refers to the number of connections it has to other nodes. On the other hand, betweenness reveals the ability of a node to act as a bridge along the shortest path. In the present microbial network analysis, the degree of distribution in female fish was lower compared to males (1.73 and 2.96). Similarly, the betweenness in female fish was lower than male fish (75.37 and 329.32). These results indicate that the microbial community in the buccal cavity of male fish has more inter-taxa associations/microbe-microbe interactions compared to that in female fish. In addition, *Staphylococcus* and *Streptococcus* had a higher degree and betweenness in the bacterial network of males compared to females.

## Discussion

To our knowledge, this is the first study that shows differences in the oral microbiome in female and male Nile tilapia that were reared in a recirculating aquaculture system. The early stages of embryonic development occur in the mouth of females, i.e., until they become hatchlings. Furthermore, fry seek shelter in the mouth of their mothers even after they start feeding on exogenous feeds (Popma and Masser, 1999; Coward and Bromage, 2000). This close association of fry and their maternal mouthbrooders indicate the importance of the oral microbiome in egg development. Moreover, the oral microbiome that is more exposed to the external environment has an indirect connection with the gut microbiome (Olsen and Yamazaki, 2019). Nevertheless, coaggregation of genetically distinct oral bacterial strains are strong, and a previous study has reported the weak interaction between oral and gut bacteria (Ledder et al., 2008). Although there is ample information about the gut microbiome of fishes, very little is known about their oral microbiome.

### Bacterial Diversity in the Buccal Cavity of Female and Male Nile Tilapia

Alpha diversity analysis revealed low differences (not significant) in species richness (191 vs. 186) and evenness (28 vs. 29.7) between females and males, while Simpson diversity was 12.2 in females and 17 in males. The non-significant differences that we observed are similar to those described during a Tenacibaculosis outbreak in Atlantic salmon (Wynne et al., 2020). Nevertheless, the authors reported that dysbiosis in the oral microbiome of the fish was due to the dominance of *Tenacibaculum* spp. (Wynne et al., 2020). Furthermore, studies on the oral microbiome of healthy human subjects (Caselli et al., 2020), adolescents suffering from anxiety and depression (Simpson et al., 2020), and patients with esophageal carcinoma (Wang et al., 2019) reported non-significant statistical differences in the microbial composition (Wang et al., 2019; Caselli et al., 2020; Simpson et al., 2020). In the present study, the fishes used were apparently healthy, and our results showed that the microbial composition (based on the proportions) in females and males was different.

### Microbial Composition in the Buccal Cavity of Female Nile Tilapia Tilts the Abundance of *Streptococcus*

The most dominant microbial phyla in the buccal cavity were Proteobacteria, Bacteroidetes, Firmicutes, Deinococcus–Thermus, Actinobacteria, and Acidobacteria. These phyla, except Deinococcus–Thermus, were also dominant in the gut and skin of Nile tilapia and Atlantic salmon (Kuebutornye et al., 2020; Sakyi et al., 2020). The presence of these dominant phyla is not affected by factors such as diet, salinity and rearing systems, but their abundances are affected by such environmental parameters (Giatsis et al., 2015; Souza et al., 2020; Yukgehnaish et al., 2020). Proteobacteria, Bacteroidetes and Firmicutes are the most dominant phyla in the oral microbiome of dolphins and sea lions (Bik et al., 2016). Proteobacteria, Bacteroidetes, Firmicutes and Actinobacteria are also dominant in the buccal cavity of humans (Zaura et al., 2009; Almeida et al., 2020; Caselli et al., 2020). At the genus level, the most abundant bacteria in the human mouth is *Streptococcus*, and in children, there exists a significant negative correlation between the counts of *S. mutans* and secretory IgA (S-IgA), pH and flow rate of saliva (Sood et al., 2014). Furthermore, the abundance of many other opportunistic pathogens such as *Pseudomonas*, *Gemella*, and *Veillonella* was lower in female fish (1.37%) compared to male fish (3.15%). The proportion of bacteria belonging to the genus *Rheinheimera* was lower in female Nile tilapia. Diketopiperazines from *Rheinheimera japonica*, isolated from marine sediments, have been reported to exert antimicrobial activity against *Bacillus subtilis*, *Enterococcus faecium*, and *Staphylococcus aureus* (Kalinovskaya et al., 2017). Furthermore, the diketopiperazine factor in another marine bacterium, *Rheinheimera aquimaris* QSI02 is efficient in controlling quorum sensing systems of *Chromobacterium violaceum* and *Pseudomonas aeruginosa* (Sun et al., 2016). These previous reports indicate the ability of the opportunistic bacteria to suppress the growth of other bacteria and the activity of host defense molecules to regulate the abundance of opportunistic bacteria such as those belonging to *Streptococcus* sp.

Although the abundances of some of the microbes were higher in males compared to females, the analysis did not detect any statistical differences in beta diversity. This finding is also similar to that observed in the human oral microbiome studies (Almeida et al., 2020; Simpson et al., 2020). In our case, we found a statistical trend in the case of the unweighted UniFrac distance, which could be linked to the near absence of *Streptococcus* bacteria in female fish.

We found that many opportunistic pathogens had significantly lower abundance in the female fish, namely *Streptococcus* with about −20 fold-change. *Streptococcus* is abundant in the human buccal cavity, and many commensal bacteria belonging to this genus play a vital role in maintaining the microbiota balance and ensuring human oral cavity health (Zaura et al., 2009; Caselli et al., 2020). Members of this genus are reactive against S-IgA in saliva, and it is known that certain species of *Streptococcus* can cause diseases in the human oral cavity and infections in the respiratory tract (Kilian et al., 1996; Zaura et al., 2009). Streptococcal infection caused by the major bacterial pathogen *Streptococcus* sp. was reported in freshwater fish such as Nile tilapia and marine fish species (Xu et al., 2007; Jantrakajorn et al., 2014), and the disease has caused significant losses in tilapia farming (Xu et al., 2007). However, a study reported that the prevalence of *Streptococcus* sp. was relatively low in nursing Nile tilapia (Jantrakajorn et al., 2014). Interestingly, in the present study, the abundance of *Streptococcus* was much lower (0.01%) in females compared to males (3.02%) (Supplementary Figure 2). Similarly, in the oral cavity of pregnant women, the abundance of *Streptococcus* and *Veillonella* was lower compared to non-pregnant women (Lin et al., 2018). Therefore, we speculate that the lower abundance of *Streptococcus* in the buccal cavity of female tilapia could be due to the mouthbrooding nature of this species. Opportunistic pathogenic members of this genus might cause egg mortality. Moreover, Streptococcosis disease can affect any stage of Nile tilapia, and one of the clinical signs is hemorrhage at the base of the mouth (Jantrakajorn et al., 2014).

There were also differences in the abundance of other pathogenic bacteria such as *Gemella* and *Veillonella*. Bacteria belonging to these 3 genera (*Streptococcus*, *Gemella*, and *Veillonella*) form biofilms in the human oral cavity (Zaura et al., 2009; Caselli et al., 2020). Interestingly in the human oral microbiome, coaggregation occurs between genetically distinct bacteria (Kolenbrander, 1988), and in children, metabolic cooperation between *Veillonella* and *Streptococcus* species occurs at the early stage of biofilm formation (Mashima and Nakazawa, 2015; Mutha et al., 2019). Furthermore, *Veillonella* was associated with many human dental diseases such as chronic periodontitis (Mashima and Nakazawa, 2015), and the presence of *Veillonella* can reduce the biofilm formation capacity of certain *Streptococcus* sp. (Mashima and Nakazawa, 2014). *Gemella* and *Streptococcus* species were found in oral plaques of patients without periodontitis (Eberhard et al., 2017), and these microbes are part of the oral microbiota in humans (Welch et al., 2019). In the present study, we found that these microbes are members of the buccal cavity of both females and males, but their abundances were different. Furthermore, the abundance of species belonging to *Streptococcus*, *S. agalactiae* was higher in the intestine of Streptococcus-infected Nile tilapia compared to healthy fish (Silva et al., 2020). Streptococci can produce hydrogen peroxide (H_2_O_2_), and it is known that while certain oxidative stress-resistant bacteria such as *Rheinheimera* sp. can benefit from H_2_O_2_ treatment, others like Verrucomicrobia may find it difficult to survive (Piel et al., 2021). In our study, we found that when ASVs of *Streptococcus* had lower abundance in the buccal cavity of female fish, Verrucomicrobia thrived. Another bacterial genus that had higher abundance in female fish was *Acinetobacter*, which is a member of microbiota in healthy human gum area (Costalonga and Herzberg, 2014), and this bacteria can be exploited for beneficial applications because of their ability in biodegradation, to synthesize high molecular weight molecules, and to enhance growth (Adegoke et al., 2012). However, it should be noted that the benefits of *Acinetobacter* are not yet exploited in aquaculture, for example, their ability to produce lipase (Adegoke et al., 2012). A study that investigated the oral bacteria in Atlantic salmon reported that the abundance of *Acinetobacter* was higher in the oral microbiome of yellow mouth disease survivors (Wynne et al., 2020). Bacteria of the genus *Acinetobacter* need low pH and nitrogen (Baumann, 1968), and the higher abundance of *Nitrospira* in female tilapia indicates the presence of nitrogen sources in the mucus of the females. In addition, Acidobacteria that are considered *K*-strategists can thrive in low pH environments (Männistö et al., 2007), and it is presumed that along with *Nitrospira*, Acidobacteria contribute to nitrification (Gülay et al., 2019). Hence, the hormonal changes that suppress appetite and reproductive functions during mouthbrooding (Das et al., 2019) could also create a conducive environment for bacteria that feeds on nitrogen. Yet another bacterial genus that had significantly higher abundance in the female Nile tilapia was Saccharibacteria. These bacteria are ultrasmall obligate parasites that lack the ability to synthesize their own amino acids and vitamins. It was reported that bacteria from this phylum parasitize other oral bacteria in humans (Bor et al., 2019; McLean et al., 2020). Furthermore, Saccharibacteria is reported to be a parasite of Actinobacteria, and this association causes slow growth of its host (Bor et al., 2020). Our results showed that the abundance of Saccharibacteria was high in the female buccal cavity, while the abundance of the *Kocuria* which belongs to the phylum Actinobacteria was lower. This could be due to the parasitic activity of Saccharibacteria. *Kocuria* is an opportunistic pathogen that was reported to be the agent of rainbow trout fry syndrome in salmonids (Pêkala et al., 2018). Interestingly, *Sphingomonas*, *Sphingobium*, *Novosphingobium*, and *Sphingopyxis* belonging to Sphingomonadaceae that are hydrocarbon degraders (Kertesz and Kawasaki, 2010) had lower abundance, and Saccharibacteria that are organic carbon sinks in hydrocarbon-fueled environments (Figueroa-Gonzalez et al., 2020) and starch degraders (Baker, 2021) had higher abundance in the buccal cavity of female Nile tilapia.

We found that the bacteria in the female buccal cavity with few potential opportunistic pathogens probably create an environment that could likely aid the host in fighting invasive pathogens such as *Streptococcus*; for example, by reducing the biofilm-forming and H_2_O_2_-producing ability of *Streptococcus*, maintaining a balance between the growth of organic and inorganic compound degraders and lipase producers. This could be a strategy adopted by the parent fish to create a stable egg incubation environment, which eventually would have an effect on the climax community of the juveniles (Krishnan et al., 2017). This climax community may have microorganisms that depend on each other via established cross-feeding strategies or other communication tactics to maintain stability over time (Díaz and Kolenbrander, 2009). However, further investigation is needed to support this hypothesis.

### Microbial Networks in the Buccal Cavity of Female Nile Tilapia Disfavor the Abundance of *Streptococcus*

Network analysis has been extensively used by biologists and computer scientists to explore interactions between entities by analyzing nodes and their connections through edges. This approach offers insight into the structure of complex inter-taxa association. In the present study, microbial network analysis was used to identify the inter-taxa association of the communities of the buccal cavities of female and male tilapia. The strong microbe-microbe interaction in male fish and the presence of more opportunists indicate the importance of cautious monitoring for the early detection of disease outbreaks in male tilapia rearing systems. It should be noted that the abundance of opportunistic pathogens is considerably higher in males, and the network analysis also indicated better microbe–microbe interactions. It was reported that opportunistic pathogens are part of the oral microbiome and their low abundance is not usually related to any disease. Nevertheless, the overgrowth of these pathogens might result in dysbiosis, which increases the risk of diseases (Radaic and Kapila, 2021).

**FIGURE 6:**
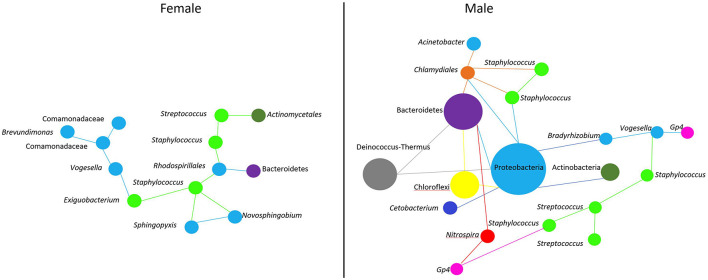
Predicted association of *Streptococcus* and *Staphylococcus* in the buccal cavity of female and male Nile tilapia. In females, we find a single cluster (Figure 5A) of microbe-microbe interaction. While in males, microbe interactions are complex (Figure 5C). Bacteria belonging to a particular phylum are color coded. The size of the nodes are based on the abundance of the ASVs.

As in any other environment, the oral cavity favors microbe-microbe interactions. Early colonizers are biofilm producers and feed on oral glycoproteins and salivary mucins (Radaic and Kapila, 2021). Around 80% of these microbes in the oral cavity of humans are represented by *Streptococcus* species (Velsko et al., 2019; Radaic and Kapila, 2021). There is growing evidence that biofilm-producing bacteria can interact physically and metabolically to form the initial biofilm community (Lamont et al., 2018). A study in lumpfish reported that a high abundance of *Tenacibaculum* on eggs can be an indication of egg mortality (Roalkvam et al., 2019). Hence, the presence of opportunistic bacteria could affect the quality of eggs and eventually the progeny. As stated earlier, *Streptococcus* spp. can produce hydrogen peroxide (H_2_O_2_), which is sufficient to kill many oral microbes, including *Staphylococcus* spp. (Jakubovics et al., 2008; Janek et al., 2016). However, it has been reported that the majority of *Staphylococcus* spp. in humans are commensal bacteria and can produce antimicrobial compounds known as bacteriocins with widely diverse activity spectra (Janek et al., 2016). *Staphylococcus*-derived bacteriocins can inhibit the action of H_2_O_2_ from *Streptococcus* spp., thereby limiting the growth of the latter in the human nasal cavity (Janek et al., 2016). The network analysis in the current study showed that *Streptococcus* spp. have a limited microbe-microbe interaction in the female buccal cavity. In the female fish-associated network, there were two *Streptococcus* spp. One (DENOVO91) that interacted with other microbes and another (DENOVO20) that did not. The interaction of *Streptococcus* (DENOVO91) in females was found in a separate cluster away from the main network (Figure 6), and in the subset we observed two *Staphylococcus* bacteria interacting with *Streptococcus.* The presence of many *Staphylococcus* ASVs compared to *Streptococcus* may indicate competition between these microbes. In contrast, in males, *Streptococcus* interacted with *Staphylococcus* and many other bacteria in the main network (Figure 6). Thus, we found that microbe-microbe interactions were less and the abundance of opportunistic bacteria was lower in the female buccal cavity. This could be due to the mouthbrooding nature of the fish to keep a suitable growth and incubation environment for the eggs.

## Conclusion

We successfully profiled the microbial communities in the buccal cavity of female and male Nile tilapia. Our results suggest that opportunistic pathogens such as *Streptococcus* are much less abundant in the female buccal cavity compared to male fish. In addition, the abundance of certain bacteria that have metabolic advantages over others was higher in female Nile tilapia. This is the first report that highlights the importance of the presence of presumed beneficial community in the oral microbiome of female Nile tilapia that are mouthbrooders.

## Data Availability Statement

The data used in this study is available at Sequence Read Archive (SRA) with accession no. PRJNA763184.

## Ethics Statement

The animal study was reviewed and approved by the Norwegian Animal Research Authority (FOTS ID 1042).

## Author Contributions

VK, JF, and YA designed the study. YA carried out the sampling, lab work, and prepared the library, analyzed the data, and wrote the manuscript. CD analyzed the data. ES performed sequencing and data generation. DA involved in initial data analyses. YA, JF, CD, and VK interpreted the data. VK, JF, and CD reviewed and edited the manuscript. All authors approved the manuscript.

## Conflict of Interest

The authors declare that the research was conducted in the absence of any commercial or financial relationships that could be construed as a potential conflict of interest.

## Publisher’s Note

All claims expressed in this article are solely those of the authors and do not necessarily represent those of their affiliated organizations, or those of the publisher, the editors and the reviewers. Any product that may be evaluated in this article, or claim that may be made by its manufacturer, is not guaranteed or endorsed by the publisher.
