# Supplementary material for: Power Play of Commensal Bacteria in the Buccal Cavity of Female Nile Tilapia
Source: Front Microbiol. 2021 Nov 16;12:773351. doi: 10.3389/fmicb.2021.773351 (PMC8636895; doi:10.3389/fmicb.2021.773351)
Supplement: Supplementary file 1 [file Data_Sheet_1.docx]

Power play of commensal bacteria in the buccal cavity of female Nile tilapia

Yousri Abdelhafiz^1^, Jorge M. O. Fernandes^1^, Erika Stefani^2^, Davide Albanese^2^, Claudio Donati^2^, Viswanath Kiron^1*^


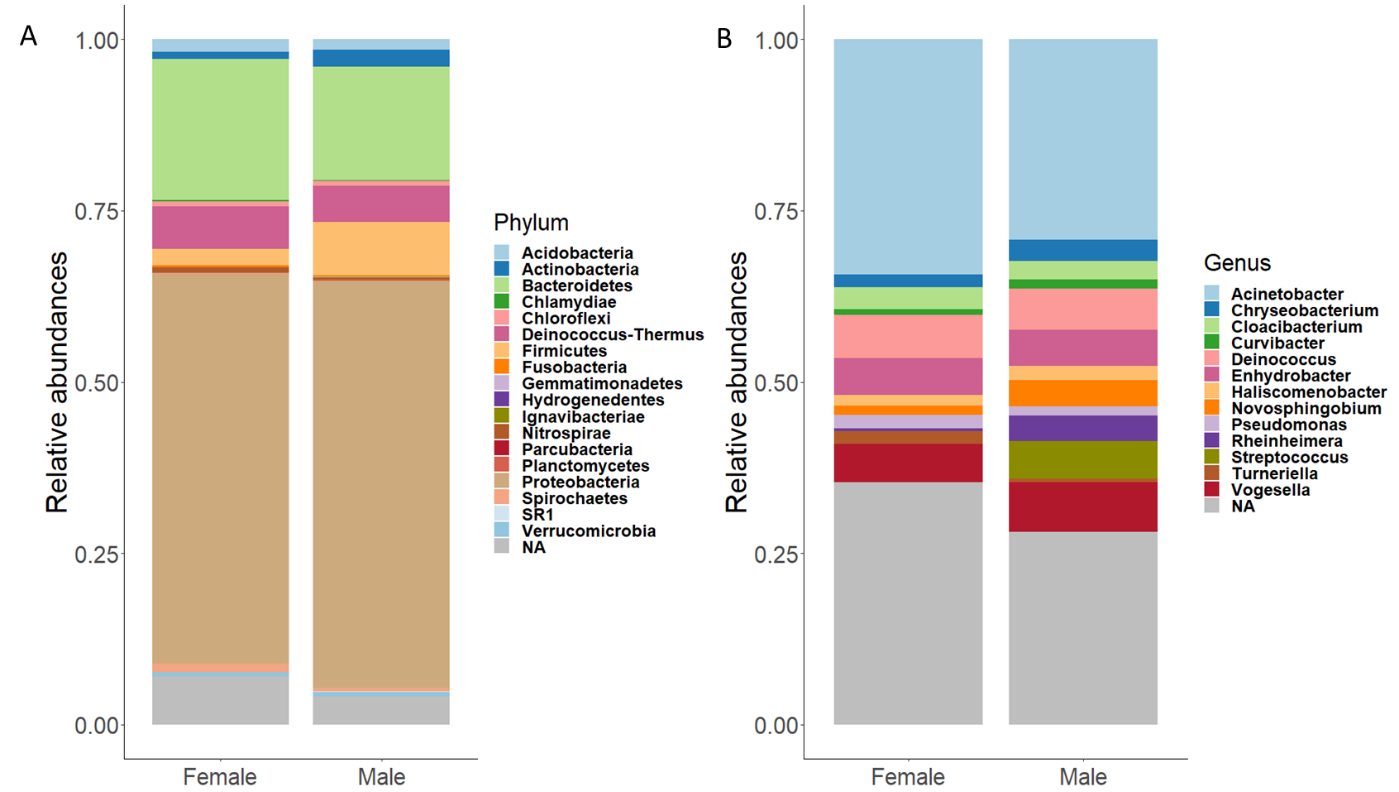


Supplementary Figure 1. The relative abundance of the microbial composition in the buccal cavity of female and male Nile tilapia. (A) Phylum level (B) Top 14 genera.


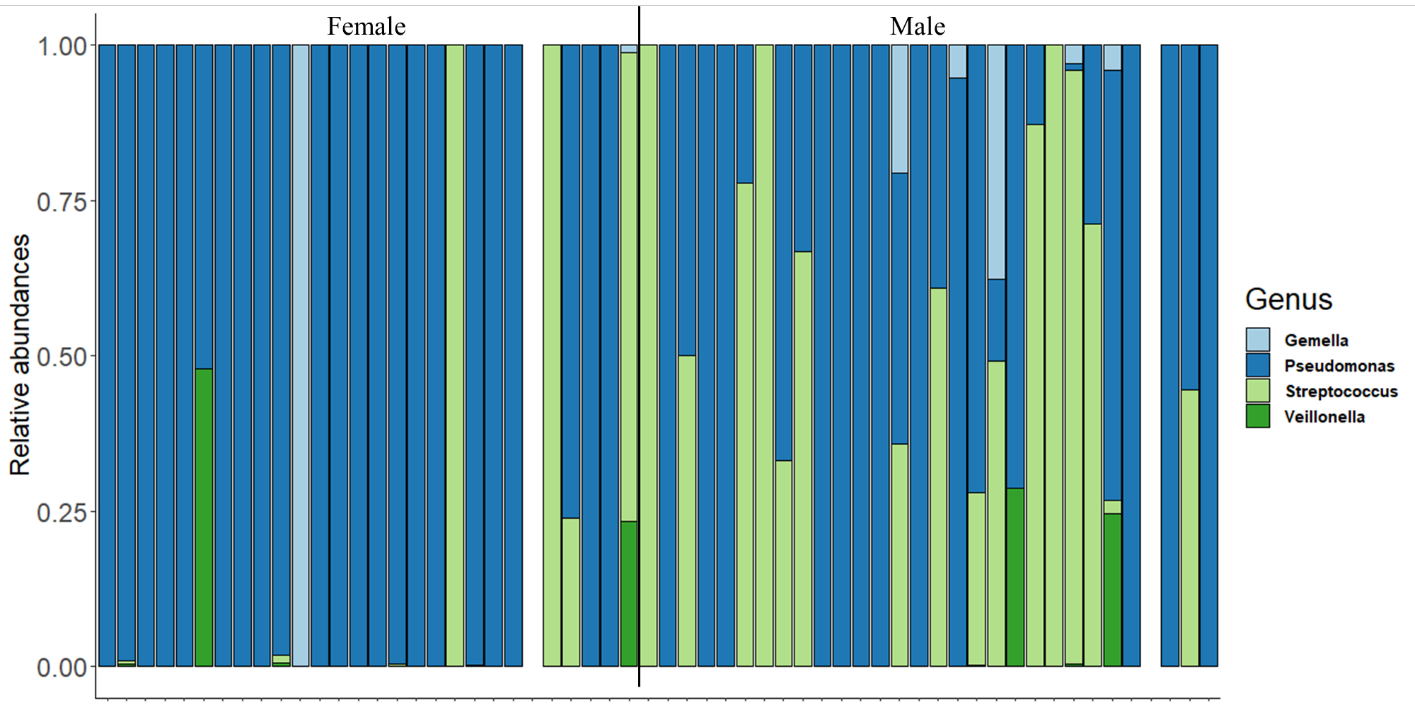


Supplementary Figure 2. Relative abundance of *Gemella*, *Pseudomonas*, *Streptococcus*, and *Veillonella* in all of the samples. Here we considered dominant and rare microbial communities, i.e. all the relevant ASVs in the data.
